# Live Attenuated Vaccines for African Swine Fever: Perspectives on Their Use and Strategic Directions

**DOI:** 10.1155/tbed/2020820

**Published:** 2026-09-18

**Authors:** Nguyen Van Diep, Pham Ngoc Doanh

**Affiliations:** ^1^ AVAC Vietnam Joint Stock Company, Hung Yen, Vietnam; ^2^ Institute of Biology, Vietnam Academy of Science and Technology, Hanoi, Vietnam, vast.ac.vn

**Keywords:** African swine fever, ASF vaccine, benefit–risk balance, biosafety, live attenuated vaccine, vaccination strategy

## Abstract

African swine fever (ASF) continues to cause substantial economic and production losses in affected countries. Although biosecurity and conventional control measures remain essential, the challenges of ASF control underscore the need for safe and effective vaccines as additional tools, particularly in endemic settings. Live attenuated vaccines (LAVs) are the most advanced ASF vaccine platform, providing the most consistent protection to date and remaining the only ASF vaccines to reach commercial deployment. Field experience with licensed products has shown encouraging safety, immunogenicity, and disease‐control outcomes. However, the introduction and spread of recombinant genotype I/II strains may reduce vaccine effectiveness and contribute to limited vaccine uptake. Together with the detection of vaccine‐like variants of uncertain origin, these issues have generated contrasting perspectives: a precautionary view focused on risks associated with replication‐competent vaccine viruses, and a benefit–risk view emphasizing the consequences of continued ASFV circulation without effective vaccination. This review contrasts these perspectives, draws lessons from other live vaccines, and frames the responsible use of ASF LAVs within a benefit–risk approach. Experience with human and veterinary LAVs shows that persistence, transmission, genetic instability, reversion to virulence, and recombination are inherent biosafety concerns of LAVs, yet such vaccines remain important when alternative platforms do not provide equivalent protection. Their use therefore requires identifying, monitoring and minimizing residual risks in relation to expected benefits. Accordingly, ASF LAVs should be avoided in ASF‐free regions but may be justified in endemic settings when licensed products are used within coordinated programs integrating adequate coverage, biosecurity, surveillance, and molecular monitoring. Future vaccine development should focus on improving safety in growing pigs and breeding animals, broadening protection against emerging ASFV strains, validating DIVA tools and developing safer next‐generation platforms, while continued reassessment ensures that vaccine use remains appropriate to epidemiological conditions, emerging evidence, and disease‐control needs.

## 1. Introduction

African swine fever (ASF) remains one of the most devastating diseases affecting domestic pigs and wild boar worldwide [1, 2], causing substantial economic losses and adversely affecting livelihoods, food security, and international trade [1–3]. Since January 2022, at least 12 countries have reported their first occurrence of ASF and 11 others its spread to previously unaffected areas, while more than 2 million animal losses were recorded worldwide by February 28, 2025, with Asia and Europe being the most affected regions [2].

Following its introduction into China in 2018, ASF spread rapidly across East and Southeast Asia, causing major losses in pig populations and substantial socioeconomic disruption [4, 5]. China subsequently strengthened disease‐control measures and implemented stringent biosecurity, particularly in large commercial production systems [6]. These measures included strict control of personnel, vehicles and materials, extensive surveillance and testing, and, in some production systems, air filtration. Together with restructuring and modernization of the pig industry, these interventions were associated with improved ASF control and the recovery of pig production and pork supply [6, 7]. However, such intensive biosecurity requires substantial infrastructure, investment, technical capacity, and sustained management, raising questions about its applicability across different production systems [6, 8]. This is particularly relevant in Southeast Asia, where smallholder, semi‐commercial, and backyard pig production with limited biosecurity remains widespread and particularly vulnerable to ASF [8]. More broadly, the diversity of pig‐production systems worldwide makes uniform implementation of stringent biosecurity challenging [3, 8]. Thus, although biosecurity, surveillance, movement control, and rapid outbreak response remain fundamental to ASF control, safe and effective vaccination may provide an important complementary tool where these measures cannot be fully implemented or are insufficient to achieve sustained disease control [2, 9, 10].

Efforts to develop an effective ASF vaccine for decades have explored various platforms, including inactivated, live attenuated, subunit, viral‐vectored, DNA, mRNA‐based, and virus‐like particle [10–13]. Among these platforms, live attenuated vaccines (LAVs), particularly genetically engineered gene‐deleted candidates, have demonstrated the most consistent protective efficacy [10–13]. Recent advances have enabled ASF LAVs to progress from experimental development to licensed commercial use in Vietnam and have subsequently been implemented under a government‐controlled program in the Philippines, while emergency use of AVAC ASF LIVE was approved in Indonesia in April 2025 [14]. Nevertheless, ASF LAVs remain the subject of debate because their ability to replicate, which contributes to strong immunogenicity and protective efficacy, also raises several biosafety concerns.

In this review, we examine the current role of LAVs in ASF control, discuss contrasting perspectives on their use, and propose future strategic directions. We first summarize their progression to commercial deployment, field experience, and the major challenges affecting their use. We then analyze contrasting perspectives on the use of ASF LAVs and draw lessons from the use of other live vaccines in human and veterinary medicine. Finally, we outline context‐specific principles for their use and identify priorities for future vaccine development.

As a narrative review, relevant evidence was identified through targeted searches of the peer‐reviewed and gray literature addressing ASF vaccine development, field use, biosafety, and vaccination strategies, as well as experience with other LAVs in human and veterinary medicine. Particular attention was given to original experimental and field studies, recent reviews, and relevant official reports, guidelines, and technical documents from veterinary authorities and international organizations. Sources were selected according to their relevance to the main themes of the review, with emphasis on evidence directly informing the safety, efficacy, field use, and risk management of ASF LAVs. No formal systematic review protocol was applied.

## 2. Current Status and Field Experience With ASF LAVs

### 2.1. LAVs as the Only Licensed and Field‐Deployed ASF Vaccine Platform

The development of effective vaccines against ASF remains one of the greatest challenges in veterinary virology, largely because of the biological complexity of ASFV and the incomplete understanding of protective immunity. ASFV has a large and complex DNA genome, with numerous genes associated with virulence and immune evasion through diverse mechanisms, including modulation of innate immune signaling, apoptosis, and other virus–host interactions [15]. While both humoral and cell‐mediated immune responses appear to contribute to protection, the correlates of protection remain poorly defined, and the specific antigens and immune mechanisms required to induce broad and durable immunity have yet to be fully established [16].

These challenges have limited the success of many ASF vaccine platforms that have been investigated. Inactivated vaccines have generally shown low protective efficacy against virulent ASFV and are therefore no longer a major priority in ASF vaccine development, while newer platforms, including subunit, viral vector, nucleic acid, and virus‐like particle vaccines, have not yet achieved consistently high and reproducible protection and remain under experimental evaluation [10–13]. Although these platforms offer improved biosafety, their protective performance has so far remained insufficient for field application. By contrast, LAVs more closely mimic natural infection and retain a broad range of viral antigens, allowing them to induce broader immune responses than other platforms. In particular, gene‐deleted LAVs have repeatedly demonstrated strong protection against homologous virulent challenge, with some candidates also providing cross‐protection against genetically distinct strains. This combination of broad antigenic stimulation and comparatively high, reproducible protective efficacy has enabled selected LAVs to advance beyond experimental evaluation to field testing, regulatory approval, and commercial use [10, 11, 13]. As a result, LAVs are currently the only ASF vaccine platform to have been licensed and deployed under field conditions [10–14].

### 2.2. Field Experience With Licensed ASF Vaccines

Practical disease control needs have accelerated the development and commercialization of ASF vaccines. Vietnam became the first country to authorize live‐attenuated ASF vaccines for commercial use in 2022 [10], including NAVET‐ASFVAC, AVAC ASF LIVE, and DACOVAC‐ASF2. In controlled challenge studies, both NAVET‐ASFVAC and AVAC ASF LIVE achieved complete protection against virulent genotype II ASFV. Subsequent limited‐scale evaluations conducted under different farm and management conditions, in which selected vaccinated pigs were experimentally challenged, reported protection rates ranging from 80% to 100% [17–19]. Because ASF vaccination was newly introduced, the Vietnamese Ministry of Agriculture and Rural Development required further large‐scale field evaluation before broader deployment. More than 600,000 doses of each vaccine were administered across multiple provinces, with regulatory monitoring reporting protective immune response rates of ~95% for NAVET‐ASFVAC across more than 140 farms and 93.4% for AVAC ASF LIVE across more than 500 farms [20–22]. For DACOVAC‐ASF2, a secondary review reported protective immune response rates of 80%–100% during large‐scale field use involving ~300,000 doses on farms managed by the manufacturer [22].

For AVAC ASF LIVE, post‐licensure surveillance data from five provinces representing different ecological regions of Vietnam included 178,767 vaccinated pigs [23–27]. Reported adverse reactions or mortality following vaccination were uncommon, ranging from 0% to 0.03%, while several localities reported no notable post‐vaccination adverse events. Serological monitoring showed 100% antibody positivity among the samples evaluated in Son La, Hai Duong, and Quang Ngai provinces (Table 1).

**Table 1:** Summary of AVAC ASF LIVE field deployment outcomes in five representative provinces of Vietnam during 2024–early 2025.

Province	Vaccinated pigs	Adverse reactions (%)	Antibody‐positive pigs (%)	Field observations
Son La (Phu Yen district)	667	0	20/20 (100%)	Vaccinated pigs remained healthy and were marketed as planned. No ASF outbreaks were reported during the observation period.
Lang Son	62,000	17 (0.03)	Not evaluated	ASF occurrence markedly decreased; 90% of outbreaks passed the 21‐day period and ASF was considered largely under control.
Hai Duong	71,150	17 (0.023) 3 deaths (0.004)	151/151 (100%)	Overall, the herds maintained normal growth and production performance. 100% vaccinated pigs were antibody‐positive, but both sentinel pigs (2/2) remained negative at 28 days post‐vaccination.
Quang Ngai	41,950	0	26/26 (100%)	Vaccinated pigs remained healthy and showed normal growth. The previous outbreaks passed the 21‐day observation period with no new outbreaks occurred.
Tien Giang	3000	0	Not evaluated	Almost all vaccinated pigs grew normally; in one herd ASF developed within 7 days after vaccination, 35 of 49 pigs were culled, 14 others remained healthy.
Total	178,767	0–0.03	197/197 (100%)	Field observations indicated favorable safety and disease‐control outcomes in the reported localities.

*Note*: *Source:* Synthesized by the authors from official 2024–2025 reports on the field use of AVAC ASF LIVE issued by provincial animal husbandry and veterinary authorities in Son La, Tien Giang, Hai Duong, Quang Ngai, and Lang Son, Vietnam [23–27]. Because provincial data were derived from field and administrative reports with non‐uniform surveillance and reporting procedures, formal confidence intervals or statistical comparisons across provinces were not considered appropriate.

Provincial reports also described positive disease control observations following vaccination. In Phu Yen District of Son La Province, repeated ASF outbreaks were reported between 2021 and 2023, but no new outbreaks were recorded after vaccination up to the time of reporting. Similarly, no new ASF outbreaks were detected during the monitoring period after the vaccination of more than 41,000 pigs from 4363 households in Quang Ngai Province. In Lang Son Province, large‐scale vaccination was associated with a substantial reduction in reported ASF occurrence, and ~90% of reported outbreaks had passed the 21‐day observation period without further reported cases.

Follow‐up observations from Tien Giang Province provided additional insight into vaccine use under high‐risk field conditions. In the Tan Phu Dong District, 30 pigs remaining after ASF‐affected animals had been removed from an outbreak herd were vaccinated and monitored for 28 days. All remained healthy and developed normally. In a 49‐pig herd in Cho Gao District, ASF was detected 7 days after vaccination, suggesting that some pigs may have already been in the incubation period at the time of vaccination or may have become infected shortly thereafter. Fourteen pigs survived the outbreak and continued to grow normally. These observations illustrate the challenges of vaccination under field conditions, where infection may be present but still clinically undetectable at the time of vaccination or may occur soon after vaccination, and reinforce the importance of careful herd assessment, strict biosecurity, and continued post‐vaccination surveillance.

Although these provincial data provide valuable real‐world evidence on vaccine use under field conditions, they were derived from passive surveillance and administrative reporting rather than a standardized sampling design. Differences in surveillance intensity, reporting completeness, data collection procedures, and the lack of uniform independent verification limit direct quantitative comparisons among provinces. Accordingly, these findings should be interpreted as descriptive real‐world field evidence rather than controlled estimates of vaccine effectiveness.

Beyond Vietnam, field trials conducted by the Philippine Bureau of Animal Industry found that AVAC ASF LIVE had a favorable safety profile and induced 100% seroconversion under field trials [28]. Although controlled challenge studies with AVAC ASF LIVE showed that 100% seroconversion at 28 days post‐vaccination was accompanied by 100% protection against virulent challenge [18], the 100% seroconversion reported in the Philippine field trial should be interpreted as an immune‐response outcome and should not be considered equivalent to a direct measure of protective efficacy. Nevertheless, the Philippine field trials remain important because they provide evidence on vaccine safety and immune responses under real‐world management conditions, consistent with the emphasis of the GF‐TADs Lomé Declaration on evaluating vaccination under field conditions [29]. Based on these findings, the Philippines subsequently authorized the controlled importation and use of AVAC ASF LIVE [28].

## 3. Emerging Challenges to ASF Vaccine Deployment

The field performance of current ASF vaccines should be interpreted against a rapidly evolving ASFV epidemiological landscape. Because the vaccines currently licensed in Vietnam were developed from genotype II strains, the promising field results reported to date largely reflect vaccine performance in regions where genotype II viruses remain predominant.

However, recent molecular epidemiological studies indicate that the ASFV population circulating in Vietnam is changing. In addition to classical genotype II strains, recombinant genotype I/II ASFV strains have been detected since 2023 [30] and appear to be increasing in prevalence. Some reports indicate that the proportion of these recombinant strains increased from ~14% in 2023 to more than 40% in 2024 [31] and accounted for more than 83% of ASFV‐positive diagnostic samples examined in several regions during 2025 [32]. Because this estimate was based on diagnostic samples rather than population‐based surveillance, it should not be interpreted as representing the true epidemiological prevalence of recombinant strains in the field.

These changes have important implications for vaccine performance. Experimental evidence indicates that cross‐protection conferred by genotype II‐based LAVs against highly virulent recombinant genotype I/II strains may be incomplete and influenced by challenge conditions. Diep et al. [33] showed that two current LAVs, ASFV‐G‐ΔMGF and ASFV‐G‐ΔI177L, did not provide complete protection against a recombinant genotype I/II strain following challenge with 10^3^ HAD_50_. More recent experimental data with the AVAC ASF LIVE vaccine showed complete protection at lower challenge doses (10–20 HAD_50_), whereas survival decreased to 40.0%–66.7% at higher doses (100–200 HAD_50_), with a two‐dose regimen showing greater protection than a single dose [34]. Together, these findings suggest that cross‐protection is possible but may be incomplete and may vary with the challenge dose, vaccination regimen, and the antigenic and genetic relatedness between vaccine and challenge strains. Therefore, disease occurrence in vaccinated herds should be interpreted together with molecular epidemiological investigations to identify the causative virus strain and avoid misleading conclusions regarding vaccine quality.

Another issue is the detection of ASFV variants resembling vaccine strains. These categories should be clearly distinguished: licensed vaccine strains are vaccine strains incorporated into vaccines authorized for commercial use by the competent regulatory authority; experimental/unauthorized vaccine strains are not approved for commercial use; vaccine‐like variants share genetic markers with vaccine strains but may differ in other genomic regions and have uncertain origins, whereas vaccine‐derived viruses have a vaccine‐strain origin confirmed by concordant molecular and epidemiological evidence. Ambagala et al. [35] reported an ASFV variant designated ASFV‐GUS‐Vietnam that contained deletions in the multigene family region and carried a *gusA* marker gene, resembling features of the ASFV‐G‐ΔMGF vaccine backbone. This variant was detected in samples collected in 2021 before the controlled deployment of ASF vaccines began in Vietnam in July 2022, so there is insufficient evidence to link it directly to licensed vaccination programs. Its origin may be associated with unauthorized or experimental virus strains circulating outside regulated settings, similar to observations reported in China where gene‐deleted ASFV strains resembling vaccine candidates were detected, although no commercial ASF vaccines had been approved [36].

One study reported a genetically distinct vaccine‐like variant (VN/sows_32005/23) in a non‐vaccinated breeding herd, which showed substantial genomic alterations, including multiple large deletions beyond those present in the related ASFV‐G‐ΔMGF vaccine backbone [37]. In another molecular surveillance study, vaccine‐like ASFVs with relatively subtle genetic differences were detected in 2.4% (6/245) of samples from non‐vaccinated farms, including 1.6% related to ASFV‐G‐ΔMGF and 0.8% related to ASFV‐G‐ΔI177L [38]. The origin, epidemiological significance, and extent of field circulation of these vaccine‐like viruses remain unclear.

Low vaccine coverage remains another major practical challenge. Since their authorization for use in 2022, ~10 million doses of ASF vaccines from AVAC JSC and NAVETCO have been produced and supplied in Vietnam. However, vaccine use has declined substantially following reports of recombinant genotype I/II viruses in Vietnam. Given Vietnam’s annual pig population of ~30 million, actual vaccination coverage therefore remains very low. By comparison, modeling studies estimated that coverage exceeding 80% would be required to substantially reduce ASFV circulation [39]. This large coverage gap limits the population‐level impact of vaccination and may lead to continued outbreaks to be incorrectly interpreted as evidence of vaccine failure.

The limited vaccine use likely reflects several interacting factors. ASF vaccines are relatively new and more expensive than many routinely used swine vaccines, and farmers may have to bear much of the vaccination cost. Differing views among scientists, veterinary authorities, and livestock‐sector stakeholders regarding vaccine safety, effectiveness, and appropriate use have also contributed to uncertainty. In addition, outbreaks occurring shortly after vaccination may reflect undetected infection before vaccination, vaccination during the incubation period, or inappropriate vaccine use rather than vaccine failure. Such events can contribute to concerns about vaccine quality or effectiveness. The emergence and circulation of recombinant or genetically distinct ASFV strains may further reinforce the perception among pig owners that vaccination is ineffective. Regulatory uncertainty may discourage vaccination. ASF control regulations largely predate vaccines, so PCR‐positive pigs may be treated as infected and subjected to culling or other control measures. Because vaccine‐derived ASFV DNA can be transiently detected in healthy vaccinated pigs without an infectious virus [40], the absence of guidance distinguishing vaccine‐strain DNA positivity from field‐virus infection may restrict movement or sale of healthy pigs, causing losses and reducing vaccine uptake. These barriers collectively restrict vaccination coverage and may substantially compromise the overall effectiveness of ASF vaccination programs.

## 4. Perspectives on the Use of ASF LAVs

The field experience and emerging challenges described above have shaped two contrasting perspectives on the use of ASF LAVs. The first, a precautionary perspective, emphasizes the biosafety risks associated with replication‐competent vaccine viruses, including persistence, transmission, genetic change, and recombination under field conditions. The second, a benefit–risk perspective, evaluates these risks in relation to the substantial production losses and broader socioeconomic consequences associated with persistent ASF circulation in the absence of effective vaccination.

### 4.1. Precautionary Perspective

The detection of vaccine‐like ASFV variants and experimental evidence of instability in some vaccine candidates have intensified concerns about the biosafety of ASF LAVs. From a precautionary perspective, their use requires particular caution because attenuated ASFV strains may persist, evolve, transmit, or recombine under field conditions, especially in endemic settings where multiple ASFV strains may co‐circulate [5, 22].

In China, gene‐deleted ASFV strains resembling vaccine candidates were detected despite the absence of approved commercial ASF vaccines, raising concerns about the unauthorized use of experimental or unlicensed strains [36]. In Vietnam, vaccine‐like variants have also been reported, although no direct link has been established between these variants and currently licensed vaccination programs [35, 37, 38]. These findings indicate that attenuated or modified ASFV strains can circulate outside regulated vaccination frameworks, potentially altering clinical presentation and complicating disease detection, control, and molecular epidemiological interpretation.

Further concerns have arisen from experimental studies examining the stability and safety of ASFV‐G‐ΔI177L. Reported findings include clinical disease and impaired semen quality in adult breeding boars, adverse reproductive effects in pregnant sows, and apparent reversion to virulence by the third in vivo passage [41, 42]. However, pregnant sows and breeding boars are outside the recommended target population of the currently available vaccine, which is intended for healthy young pigs. Moreover, the serial‐passage findings differ from those of Tran et al. [17], who observed no clinical reversion to virulence after five passages using the master seed virus. Differences in virus preparation, passage history, or experimental design may therefore have contributed to the divergent outcomes. These studies do not necessarily demonstrate that the licensed vaccine is unsafe when used according to its approved indication, but they highlight the importance of strict adherence to product specifications and the need for specific reproductive safety data before use is extended to breeding animals.

The precautionary perspective is therefore scientifically justified because it identifies potential biological risks associated with ASF LAVs and emphasizes the need for caution and rigorous evaluation before broader deployment [5, 35–38, 41–43].

### 4.2. Benefit–Risk Perspective

A benefit–risk perspective evaluates ASF vaccination by weighing the expected benefits and risks of vaccination against the consequences of not vaccinating and continued disease circulation. This balance depends on disease‐control needs, epidemiological conditions, production systems, and the broader consequences of ASF, including animal health, production, and socioeconomic impacts [44, 45].

The 2026 WOAH [45] framework explicitly illustrates that both vaccination and non‐vaccination may entail benefits and risks. Vaccination may reduce clinical disease, mortality, production losses, and disruption to farming activities but may also involve risks such as vaccine‐virus shedding, adverse events, insufficient vaccination coverage, and lower‐than‐expected efficacy. Conversely, not vaccinating may avoid the use of a vaccine that is poorly matched to circulating ASFV strains but increases reliance on surveillance and strict biosecurity, which may not be feasible in all production systems. Thus, the relative consequences of both options should be considered within the relevant epidemiological and production context.

This balance may therefore differ substantially between settings. In ASF‐free regions, the expected benefits of introducing replication‐competent LAVs may be limited relative to their potential biosafety risks, and routine use should generally be avoided. In contrast, in endemic regions with recurrent outbreaks, continued ASFV circulation may impose substantial animal health, production, and socioeconomic consequences [44, 45]. As emphasized by Penrith [46], a safe and effective ASF vaccine may provide important advantages for disease management but should complement, rather than replace, effective biosecurity.

This context‐dependent benefit–risk perspective has been increasingly recognized in the scientific literature [10, 11, 13, 46, 47] and is also reflected in international guidance [2, 9, 29, 44, 45]. WOAH emphasizes consideration of vaccine quality, safety, and efficacy together with the epidemiological situation, target population, and intended use [2, 44, 45]. FAO similarly promotes responsible vaccine use within broader ASF control strategies [9], while the GF‐TADs Lomé Declaration supports appropriately regulated vaccination based on vaccine safety, efficacy, and quality [29].

These frameworks reinforce the principle that safety and efficacy should be considered jointly. A vaccine providing substantial protection but presenting unacceptable biological risks would not be suitable, whereas a vaccine with a favorable safety profile but insufficient protective efficacy would offer limited disease‐control value. Because LAVs remain live and replication‐competent and retain certain biological risks [48], the key question is whether these residual risks are adequately characterized and can be managed to an acceptable level relative to the expected benefits under the intended conditions of use [47]. Accordingly, a benefit–risk perspective supports neither indiscriminate deployment nor categorical rejection of ASF LAVs. The central question is therefore which vaccines should be used, where, in which pig populations, and under what conditions their expected benefits outweigh their residual risks.

## 5. Experience With Other LAVs

### 5.1. Biosafety Risks Inherent to Replication‐Competent LAVs

The same biological feature that makes LAVs immunologically effective also explains their biosafety limitations. Because the vaccine virus remains replication‐competent, it may persist in the host, be shed, transmit to susceptible contacts, or undergo genetic changes during replication. These events are not unique to ASF vaccines; they are intrinsic concerns for LAVs used in both human and veterinary medicine [48–75].

#### 5.1.1. Evidence From Veterinary LAVs

In veterinary medicine, modified live vaccines for porcine reproductive and respiratory syndrome (PRRS) provide one of the most extensively documented examples of biosafety concerns associated with replication‐competent attenuated vaccines. Recombination between vaccine and field strains, as well as between different vaccine strains, has been repeatedly reported [50–54]. Reviews by Li et al. [53] and Wang and Feng [54] identified at least 16 vaccine–field recombinant isolates and six vaccine–vaccine recombinants, some of which showed increased pathogenicity or were associated with severe disease outbreaks. Other reported concerns include persistence of vaccine virus, prolonged viremia, shedding, transmission to naïve pigs, and transplacental infection [53, 54] (Table 2).

**Table 2:** Major biosafety concerns associated with live attenuated vaccines for veterinary viral diseases.

Disease	Virus type	Reported event	Reported strain or example	References
PRRS	RNA	Vaccine–field and vaccine–vaccine recombination; persistence, shedding, and transmission of vaccine virus	At least 16 vaccine–field recombinant isolates and six vaccine–vaccine recombinants have been reported; some recombinant strains showed increased pathogenicity or were associated with severe disease outbreaks. Vaccine virus persistence, shedding, horizontal, and vertical transmission have also been documented.	[50–54]
ILT	DNA	Reversion to virulence; vaccine–vaccine recombination	CEO ILTV vaccines can regain virulence; virulent recombinant ILTV strains arose from recombination among SA2, A20, and Serva vaccine strains.	[55–57]
IB	RNA	Vaccine‐field recombination	Recombinant IBV CK/CH/SCMY/160315 involved H120‐, 4/91‐, and LDT3‐A‐related vaccine strains and field strains.	[58]
LSD	DNA	Vaccine‐derived recombinant virus	Recombinant vaccine‐derived or vaccine‐like LSDV strains, including Saratov/2017 and Udmurtiya/2019, were experimentally shown to transmit between cattle in the absence of insect vectors.	[59–61]
BTV	RNA, segmented	Reassortment	Live attenuated BTV vaccine strains repeatedly reassorted with field strains during European outbreaks, contributing to genetic, and potentially phenotypic variability.	[62]
AHS	RNA, segmented	Reversion to virulence; reassortment	AHS outbreaks in South Africa were caused by virulent revertants of AHSV type 1 live attenuated vaccine and reassortants containing genome segments derived from AHSV type 1, 3, and 4 vaccine strains.	[63]
BVD	RNA	RNA recombination between vaccine and persistent field virus	Cytopathogenic BVDV strains, including cp BVDV 1741 and CP 4584, arose through recombination between persistent pestiviruses and vaccine strains.	[64]
IBD	RNA, segmented	Reassortment	Natural reassortant IBDV Bpop/03 and Central European reassortants with vvIBDV‐derived segment A/VP2 and classical attenuated D78‐like segment B/VP1 were reported.	[65, 66]
MDV	DNA	Vaccine‐field recombination	Natural recombinant MDV strains, including HC/0803, CH/10, SY/1219, DH/1307, DH/1504, and Hrb/1504, arose from recombination between CVI988/Rispens vaccine and virulent field MDV strains.	[67]
CDV	RNA	Reversion to virulence; vaccine‐origin infection	The attenuated CDV Rockborn strain reverted to virulence experimentally, and vaccine‐origin CDV infection was reported in a 14‐week‐old puppy.	[68, 69]

Comparable biosafety concerns have been documented in several other veterinary viral diseases, including infectious laryngotracheitis (ILT), infectious bronchitis (IB), lumpy skin disease (LSD), bluetongue (BTV), African horse sickness (AHS), bovine viral diarrhea (BVD), infectious bursal disease (IBD), Marek’s disease (MDV), and canine distemper (CDV). These reports include reversion to virulence, circulation of vaccine‐derived viruses, recombination, and reassortment (Table 2). Notably, such events have been documented for LAVs against both RNA and DNA viruses, indicating that these biosafety risks are not restricted to a particular viral genome type.

#### 5.1.2. Evidence From Human LAVs

Although this review focuses primarily on ASF vaccination in the field of veterinary medicine, biosafety concerns associated with LAVs are not unique to veterinary medicine. Similar issues, including vaccine‐virus shedding, transmission, genetic evolution, and the potential recovery of virulence, have also been documented or considered for several live vaccines used in humans. The following two examples are presented to illustrate these concerns rather than to provide a comprehensive review of human live vaccines.

Oral poliovirus vaccine (OPV) provides the most well‐documented example. OPV strains can replicate in the intestine, be shed, and transmitted to susceptible contacts. During prolonged circulation in populations with insufficient immunity, these viruses may accumulate genetic changes and regain neurovirulence, resulting in the emergence and spread of circulating vaccine‐derived polioviruses [70–72]. Live‐attenuated rotavirus vaccines provide another example. Vaccine strains are commonly shed after vaccination, and vaccine‐derived strains, occasional transmission, and genetic changes, including recombination or reassortment events, have been detected in clinical samples [73–75].

Taken together, these examples show that persistence, transmission, and genetic change are not exceptional events unique to ASF LAVs but recurring considerations for replication‐competent vaccines. Their occurrence, however, does not by itself determine whether a live vaccine remains suitable for use; this depends on the magnitude of the risk and the benefits that the vaccine provides in controlling the disease.

### 5.2. Continued Use of LAVs Despite Their Biosafety Risks

Despite the biosafety concerns discussed above, LAVs continue to be widely used in both human and veterinary medicine because their immunological and epidemiological benefits often outweigh their potential risks. In many diseases, alternative vaccine platforms with improved biosafety profiles have not been able to fully replace LAVs because they may induce weaker, less durable, or less comprehensive protective immunity. This is particularly relevant for complex viral diseases in which effective protection requires broad immune responses, including strong cellular immunity.

The OPV provides a representative example in human medicine. Although OPV‐derived viruses may emerge in under‐immunized populations, OPV played a central role in global poliovirus control because of its high effectiveness and ease of administration. The withdrawal of an OPV component was considered only after the corresponding wild poliovirus serotype had been eradicated. For example, following the global eradication of wild poliovirus type 2, this component was removed through the coordinated switch from trivalent to bivalent OPV in 2016 [76]. This experience illustrates that the use of a LAV should be guided by its risk–benefit balance in the prevailing epidemiological context rather than rejected solely because it carries inherent biosafety risks.

A similar pattern can be observed in veterinary medicine. In PRRS, LAVs remain the cornerstone of vaccination programs despite well‐recognized biosafety concerns. Currently, 17 commercial PRRS LAVs are used worldwide, including six PRRSV‐1 and 11 PRRSV‐2 vaccines, whereas only a limited number of inactivated PRRS vaccines are available, some of which are no longer marketed or are restricted to specific regions. Although considerable progress has been made in the development of next‐generation PRRS vaccines, including subunit, DNA, viral‐vectored, virus‐like particle, RNA, and peptide vaccines, these platforms remain largely under development, and none has been reported to be commercially available for routine PRRS vaccination. Consequently, LAVs continue to be the primary vaccine platform for PRRS control because they induce stronger humoral and cell‐mediated immune responses and provide better field protection than other available or experimental vaccine platforms [53, 54].

Similarly, in the control of ILT in poultry, viral vector vaccines were expected to replace LAVs because of their improved biosafety profile. However, field experience has shown that their effectiveness in disease control has not met expectations [56]. Thus, despite their recognized biosafety limitations, LAVs continue to play an important role in ILT control.

These examples show that the continued use of LAVs is not based on an absence of biosafety concerns but on their ability to provide disease‐control benefits that alternative platforms have not always been able to match. The relevant question is therefore not whether a live vaccine carries biological risk but whether that risk remains acceptable in relation to the protection it provides and the consequences of leaving the disease insufficiently controlled. This balance may change over time as the disease epidemiology evolves and safer alternatives become available.

## 6. Lessons From Other Live Vaccines and Implications for ASF LAVs

### 6.1. Lessons From the Use of LAVs

**Figure 1:**
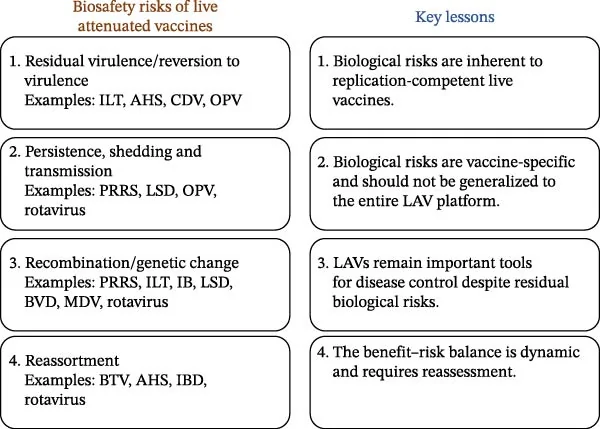
Biosafety risks of live attenuated vaccines and key lessons relevant to ASF LAVs.

Although these diseases differ from ASF in epidemiology and transmission, experience with LAVs used against them highlights the biological characteristics and biosafety risks relevant to replication‐competent ASF LAVs. Several general lessons can therefore be drawn from the use of live vaccines in both human and veterinary medicine, as summarized in Figure 1.

1. Lesson 1: Biological risks are inherent to replication‐competent live vaccines. Because LAVs retain the ability to replicate, potential risks may include residual virulence, persistence and shedding, horizontal or vertical transmission, genetic instability, recombination, and, for segmented viruses, reassortment.
2. Lesson 2: Biological risks are vaccine‐specific and should not be generalized to the entire LAV platform. The types and severity of these risks vary among vaccines. Each vaccine should therefore be evaluated on its own characteristics and supporting evidence.
3. Lesson 3: LAVs may remain important disease‐control tools when their expected benefits outweigh the residual biological risks under the intended conditions of use.
4. Lesson 4: The benefit–risk balance is dynamic and requires reassessment. The acceptability of a LAV may change with disease epidemiology, circulating viruses, new safety and effectiveness data, and available alternatives.

### 6.2. Application to ASF LAVs: A Context‐Dependent Benefit–Risk Approach

Applied to ASF LAVs, these lessons support neither premature adoption nor categorical rejection of the platform. LAVs have so far demonstrated the most consistent protective efficacy among ASF vaccine platforms and are the only platform to have progressed to a licensed field use [10–13]. However, their replication competence also introduces biological risks that require careful evaluation. Each vaccine should therefore be assessed on the basis of product‐specific evidence of quality, safety, efficacy, and genetic stability, together with the epidemiological and production context in which it is intended to be used [2, 44, 45].

**Figure 2:**
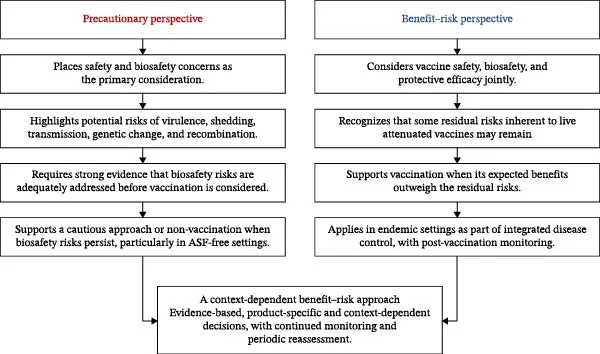
Precautionary and benefit–risk perspectives supporting a context‐dependent approach to ASF vaccination.

In the context of ASF LAVs, the precautionary and benefit–risk perspectives discussed above can be applied as complementary lenses for evaluating vaccine use (Figure 2). The precautionary perspective places safety and biosafety concerns as the primary consideration and highlights potential risks associated with residual virulence, shedding, transmission, genetic change, and recombination. Under this perspective, strong evidence that these biosafety risks are adequately addressed is required before vaccination is considered. It therefore supports a cautious approach and may favor non‐vaccination when biosafety risks persist, particularly in ASF‐free settings. In contrast, the benefit–risk perspective considers safety and protective efficacy together and recognizes that some residual risks inherent to live vaccines may be acceptable when they are sufficiently characterized, manageable, and outweighed by the expected benefits of vaccination. This perspective is particularly relevant in endemic settings, where vaccination may form part of integrated disease control, together with appropriate biosecurity and post‐vaccination monitoring. Rather than being mutually exclusive, these perspectives converge on a context‐dependent approach in which decisions should be evidence‐based, product‐specific, and adapted to the prevailing epidemiological and production context, with continued monitoring and periodic reassessment [2, 44, 45].

In practice, where removal of infected animals or herds remains an important control measure, the relative roles of removal and vaccination should be considered in light of transmission intensity, timeliness of detection, biosecurity capacity, and the feasibility of movement control, depopulation, and compensation [9, 45]. Vaccination may be favored when circulating strains remain adequately covered, product safety and genetic stability are supported by evidence, vaccine‐associated adverse events and transmission remain acceptably low, and adequate surveillance is in place. Conversely, substantial strain mismatch, important safety signals, persistent vaccine‐virus transmission, or evidence of genetic instability would shift the balance toward greater precaution, restriction, or discontinuation pending reassessment [45]. The benefit–risk balance should therefore be regarded as dynamic and periodically reassessed as epidemiological conditions, vaccine performance, and new evidence emerge.

Practical capacity must also be considered. In resource‐constrained smallholder systems, both biosecurity and vaccination programs may face implementation limitations [77]. Vaccination and biosecurity should therefore be regarded as complementary rather than competing approaches. Modeling by Barongo et al. [78] predicted greater reductions in ASF‐related pig mortality when vaccination was combined with enhanced biosecurity than when either intervention was applied alone. Thus, responsible ASF LAV use requires evidence‐based, product‐specific decisions, together with continued monitoring and periodic reassessment.

Consistent with the WOAH framework, which considers the relative benefits and risks of both vaccination and non‐vaccination [45], ASF LAV use should be determined according to the prevailing epidemiological and production context. Applied to Vietnam, the available evidence supports a context‐dependent rather than uniform approach to vaccine use. Regulatory evaluations and subsequent field reports have generally supported a favorable safety profile in vaccinated pigs within the indicated target population, together with strong immune responses and demonstrated protection against genotype II ASFV [17, 18, 21, 23–27]. However, the increasing detection of recombinant genotype I/II ASFVs [30–32], together with evidence of incomplete protection against these recombinant strains [33, 34], indicates that vaccine effectiveness cannot be assumed uniformly across epidemiological settings. Another consideration is the detection of vaccine‐like ASFVs. The origin of these variants remains uncertain; some were detected before vaccine use or in unvaccinated herds [35, 37], and their epidemiological significance and extent of field circulation remain unclear. These findings therefore underscore the importance of rigorous post‐vaccination surveillance. Taken together, the available evidence supports context‐dependent ASF vaccine use where circulating strains remain adequately covered, while emphasizing the need for molecular surveillance, biosecurity, post‐vaccination monitoring, and periodic reassessment as the epidemiological situation evolves.

## 7. Strategic Directions for ASF Vaccination

**Figure 3:**
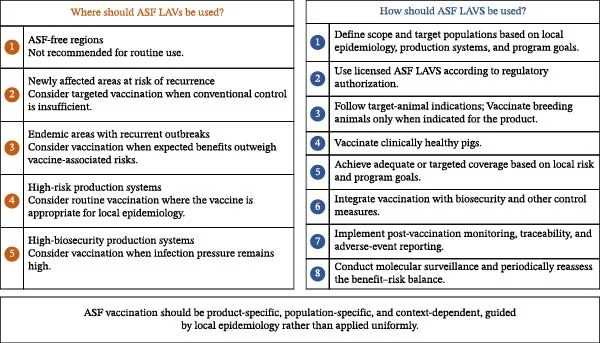
Strategic directions for ASF vaccination: where and how ASF LAVs may be used.

Translating the context‐dependent benefit–risk approach into practice requires determining where ASF LAVs may be appropriate and how they can be used to maximize disease control benefits while minimizing vaccine‐associated risks (Figure 3).

### 7.1. Where Should ASF LAVs Be Used?

ASF vaccination strategies should be adapted to the epidemiological and production context rather than applied uniformly across all settings. The benefit–risk balance may differ among ASF‐free areas, newly affected areas, endemic regions, high‐risk production systems, and production systems with strong biosecurity. Accordingly, decisions on ASF LAV deployment should consider both the local epidemiological situation and the risk of virus exposure within different production systems. Table 3 summarizes a context‐specific approach to ASF LAV use.

**Table 3:** Proposed context‐specific approach to the use of live attenuated ASF vaccines.

Epidemiological setting	Recommended approach	Rationale and key safeguards
ASF‐free areas without active outbreaks	Routine vaccination is not recommended.	The primary objective is to prevent virus introduction and maintain disease‐free status. Priority should remain on biosecurity, movement and import controls, surveillance, and rapid outbreak response.
Newly affected areas at risk of recurrence	Consider targeted vaccination when conventional control measures alone are insufficient.	Stamping‐out, movement control, surveillance, and biosecurity should remain primary measures. Vaccination may be considered where the risk of continued or recurrent outbreaks cannot be adequately controlled by these measures alone.
Endemic areas with recurrent outbreaks	Consider vaccination when expected benefits outweigh vaccine‐associated risks.	Recurrent outbreaks and persistent disease impact may increase the need for vaccination as an additional control measure. Deployment should be based on local epidemiology and integrated with surveillance, biosecurity, and other control measures.
High‐risk production systems	Consider routine vaccination where exposure risk is persistently high and the vaccine is appropriate for local epidemiology.	Backyard, semi‐intensive, free‐ranging, or other high‐risk systems may experience repeated virus exposure and may have limited capacity to maintain strict biosecurity. Vaccination should be integrated with farmer training, surveillance, reporting, and other control measures.
High‐biosecurity production systems	Consider vaccination when infection pressure remains high.	Strong biosecurity may substantially reduce exposure risk, and vaccination may therefore not always be necessary. However, additional protection may be justified where high infection pressure persists in the surrounding environment or epidemiological situation.

### 7.2. How Should ASF LAVs Be Used?

Once vaccination has been considered appropriate for a given epidemiological setting, ASF LAVs should be implemented through a coordinated, evidence‐based risk management framework consistent with applicable WOAH guidance [9, 44, 45, 79]. The following practical principles are proposed:

1. Define the geographical scope and coordinate implementation. Vaccination areas and target populations should be defined according to local epidemiology, circulating ASFV strains, production systems, risk of exposure, and program objectives [9, 45, 79]. Implementation should be coordinated among competent authorities, veterinary services and relevant stakeholders to ensure consistent application and monitoring of the vaccination strategy [45, 79].
2. Use only licensed and quality‐controlled vaccines. Vaccines should be authorized by the competent regulatory authority and supported by adequate evidence of quality, safety, efficacy, and genetic stability [44, 45, 79]. Unauthorized or inadequately characterized vaccine strains should not be introduced into commercial pig populations. Vaccine storage, transport, and administration should follow approved product instructions [79].
3. Follow the approved target animal indication. Vaccines should be used only in animal populations for which adequate product‐specific safety and efficacy data and regulatory approval are available. Vaccine selection should take account of factors such as age, health status, production type, and physiological status of the target population [44, 45]. Use in breeding populations should therefore be limited to products for which appropriate safety data and regulatory approval for such animals are available.
4. Vaccinate only clinically healthy pigs. Vaccination should be limited to animals that meet the approved eligibility criteria and show no clinical signs of illness, including signs suggestive of ASF [44, 45, 79]. Clinically ill pigs should not be vaccinated until they have recovered and met the eligibility criteria. Pigs or herds suspected of ASF should not be vaccinated until diagnostic testing and epidemiological investigation have been completed, and ASF has been excluded. Because vaccination is preventive rather than therapeutic, pigs with suspected or confirmed ASF should be managed according to established outbreak‐control procedures rather than vaccinated.
5. Achieve sufficiently high and strategically targeted vaccination coverage. Vaccination coverage should be planned according to baseline ASFV transmission, expected vaccine effectiveness, population structure, and program objectives [45, 79]. Because complete immunization of the target population may not be achievable, programs should define the level of coverage needed to achieve their intended population‐level effect [79]. A modeling study of small‐scale farms in Vietnam suggested, under its model assumptions, that vaccination of at least 80% of pigs on each farm would be required to effectively reduce ASFV transmission [39].
6. Integrate vaccination with established ASF control measures. Vaccination should complement, rather than replace, biosecurity, surveillance, movement control, and other established measures and should be implemented as part of a coordinated ASF prevention and control program [9, 45, 79]. The relative contribution of vaccination and other control measures should be adapted to the local epidemiological situation and program objectives.
7. Establish post‐vaccination monitoring and pharmacovigilance. Post‐vaccination monitoring should assess vaccine safety, field effectiveness, and overall program performance [45, 79]. Accurate records should be maintained for vaccine products, batch numbers, vaccination dates, with appropriate identification of vaccinated animals or herds where feasible [45, 79]. Suspected adverse events, unexpected disease, or apparent vaccine failures should be investigated to distinguish field infection, inadequate protection, vaccine‐related effects, and other causes.
8. Maintain molecular surveillance and periodically reassess the program. Molecular surveillance should monitor circulating ASFV strains and detect changes that could affect vaccine performance. Where appropriate and technically available, validated vaccine‐specific or DIVA molecular assays should be used to distinguish vaccine strains from field viruses [45]. Because vaccine‐strain ASFV DNA may be detected transiently after vaccination, PCR‐positive findings should be interpreted together with vaccination history and, when necessary, further characterized using vaccine‐specific molecular testing or sequencing [40, 45]. Whole‐genome sequencing should be considered when epidemiologically indicated to investigate apparent vaccine failure, persistent virus circulation, suspected reversion to virulence, recombination, or other relevant genetic changes [45]. Surveillance and post‐vaccination findings should inform periodic reassessment of vaccine choice, target populations, and vaccination strategies as epidemiological conditions and evidence change [45, 79].

These post‐vaccination monitoring and molecular surveillance activities should be proportionate to epidemiological risk, program objectives, and available technical and financial capacity. Core requirements for vaccine safety and essential surveillance should be maintained, while resource‐intensive investigations, particularly whole‐genome sequencing, should be prioritized for specific epidemiological or safety concerns. Economic feasibility and available resources should also be considered when designing and reassessing vaccination programs [9, 45].

### 7.3. Priorities for Future ASF Vaccine Development

In the longer term, ASF vaccine development should focus on improving safety and broadening protection against the genetic diversity of circulating ASFV, including recombinant genotype I/II variants. For LAVs, the priorities include improving genetic stability, reducing persistence and transmissibility, minimizing the potential for reversion or recombination, and strengthening reproductive safety. Broader use across different pig populations will also require specific safety evidence in breeding animals.

The development and validation of reliable DIVA approaches should remain a priority. WOAH notes that gene‐deleted LAVs can be differentiated from wild‐type ASFV by molecular methods, although standardized population‐level DIVA tests are not yet available [44, 45]. Molecular DIVA provides vaccine‐specific discrimination but requires detectable viral DNA, whereas serological DIVA may be better suited to large‐scale surveillance but is more difficult to develop because deletion of candidate marker genes or proteins may affect vaccine replication or protective efficacy [80, 81]. Given the genetic and antigenic complexity of ASFV, no single DIVA approach is likely to be suitable for all vaccines and surveillance purposes. DIVA strategies should therefore be tailored to vaccine design and surveillance objectives.

Parallel development of next‐generation technologies, including subunit, viral vector, DNA, mRNA, and virus‐like particle vaccines, should continue. These platforms may reduce biosafety concerns associated with replication‐competent vaccines, but achieving strong, durable, and reproducible protection against ASFV remains a major challenge. Future development should therefore prioritize broad and consistent protective immunity, with practical use considered when these platforms demonstrate meaningful and reproducible protection.

Future ASF vaccine development is therefore likely to require a dual‐track approach: continued optimization of LAVs for near‐term disease control, alongside the development of safer next‐generation platforms for long‐term control and potential eradication.

## 8. Conclusions

Scientific evidence and experience with other live vaccines show that replication‐competent LAVs may carry residual biological risks, but these risks should be considered in relation to the protection provided and the consequences of uncontrolled disease. ASF LAVs should therefore be assessed on a product‐specific, evidence‐based benefit–risk basis rather than prematurely accepted or categorically rejected as a platform. The appropriate balance depends on the epidemiological context. In ASF‐free settings, routine use of replication‐competent vaccines should generally be avoided, whereas in endemic settings, licensed LAVs with demonstrated safety and efficacy may provide an important additional control tool when used in appropriate populations and integrated with biosecurity, surveillance, and other established measures. Continued monitoring and reassessment are essential as circulating viruses, vaccine performance, and epidemiological conditions change.

Future development should follow a parallel pathway: continued improvement of LAVs in safety, genetic stability, cross‐protection, and DIVA capability, together with the development of safer next‐generation platforms. A progressive transition from LAVs to alternative platforms should be driven by evidence that these platforms provide comparable or superior protective efficacy, together with meaningful safety and operational advantages. Until such evidence is available, vaccine selection should remain based on the demonstrated benefit–risk profile of individual products rather than on the vaccine platform alone.

## Author Contributions

Nguyen Van Diep conceived the idea. Nguyen Van Diep and Pham Ngoc Doanh prepared the initial draft and revised the manuscript.

## Funding

This work received no specific research funding.

## Disclosure

All the authors contributed to the final revision of the article and approved the submitted version.

## Ethics Statement

Ethical approval was not required for this review article because no experiments were conducted. All information included in this review was derived from previously published studies.

## Conflicts of Interest

Nguyen Van Diep is an employee of AVAC Vietnam Joint Stock Company, which manufactures AVAC ASF LIVE. The other author declares no conflicts of interest.

## Data Availability

Data sharing is not applicable to this article because no datasets were generated or analyzed during the current study.
